# Curcumin: A Natural Warrior Against Inflammatory Liver Diseases

**DOI:** 10.3390/nu17081373

**Published:** 2025-04-18

**Authors:** Olga Obrzut, Aleksandra Gostyńska-Stawna, Karolina Kustrzyńska, Maciej Stawny, Violetta Krajka-Kuźniak

**Affiliations:** 1 Department of Pharmaceutical Biochemistry, Poznan University of Medical Sciences, Rokietnicka 3, 60-806 Poznan, Poland; onapierala@ump.edu.pl; 2 Department of Pharmaceutical Chemistry, Poznan University of Medical Sciences, Rokietnicka 3, 60-806 Poznan, Poland; agostynska@ump.edu.pl (A.G.-S.); mstawny@ump.edu.pl (M.S.); 3 Department of Inorganic and Analytical Chemistry, Poznan University of Medical Sciences, Rokietnicka 3, 60-806 Poznan, Poland; kustrzynska@gmail.com; 4 Doctoral School, Poznan University of Medical Sciences, Bukowska 70, 60-812 Poznan, Poland

**Keywords:** inflammation, curcumin, liver, IFALD, MASLD

## Abstract

Curcumin (CUR), a bioactive compound found in turmeric, has garnered attention for its potential anti-inflammatory properties and impact on liver health. Numerous studies suggest that CUR may be crucial in mitigating liver inflammation. The compound’s anti-inflammatory effects are believed to be attributed to its ability to modulate various molecular pathways involved in the inflammatory response. Research indicates that CUR may suppress the activation of inflammatory cells and the production of pro-inflammatory cytokines in the liver. Additionally, it has been observed to inhibit the activity of transcription factors that play a key role in inflammation. By targeting these molecular mechanisms, CUR may help alleviate the inflammatory burden on the liver. Moreover, CUR’s antioxidant properties are thought to contribute to its protective effects on the liver. Oxidative stress is closely linked to inflammation, and CUR’s ability to neutralize free radicals may further support its anti-inflammatory action. While the evidence is promising, it is essential to note that more research is needed to fully understand the precise mechanisms through which CUR influences liver inflammation. Nevertheless, these findings suggest that CUR could be a potential therapeutic agent in managing liver inflammatory conditions. In this review, we explore the potential impact of CUR on inflammation, highlighting the key mechanisms involved, as reported in the literature.

## 1. Introduction: Hepatic Inflammation

Inflammation is a complex response to various insults, such as infections, toxins, and metabolic disorders. Hepatic inflammation can be triggered by viral infections (e.g., hepatitis B and C viruses), intestinal failure-associated liver disease (IFALD), metabolic dysfunction-associated steatotic liver disease (MASLD), alcoholic liver disease, autoimmune hepatitis, and metabolic disorders. These conditions can initiate an immune response that, if unresolved, leads to chronic inflammation. Chronic inflammation in the liver can lead to more severe conditions, including liver fibrosis, cirrhosis, and hepatocellular carcinoma [1]. Macrophages and T lymphocytes undoubtedly play a key role in hepatitis. Macrophages and Kupffer cells release signaling molecules, such as cytokines and chemokines, which attract other immune cells to the inflamed area. Macrophages also help in the clearance of damaged cells and tissues, contributing to the resolution of the inflammatory process. Additionally, they regulate the immune response by presenting antigens to other immune cells, influencing the activation and function of T lymphocytes. In turn, T lymphocytes contribute to the regulation and execution of the immune response during inflammation, helping the body defend itself against infections and maintain tissue integrity [2].

Inflammation is intricately linked to signaling pathways, with nuclear factor-kappa B (NF-κB) playing a central role in this connection. NF-κB is a transcription factor that regulates the expression of genes involved in the immune response, including those responsible for inflammation. Various stimuli activate receptors on cell surfaces when tissues are damaged or infected. This activation triggers a signaling cascade that ultimately activates NF-κB, associated with its translocation to the cell nucleus, and promotes the expression of pro-inflammatory genes, such as cytokines, chemokines, and adhesion molecules.

Inflammasomes are molecular complexes central to the inflammatory response. These complexes act as sensors within cells, triggered by signals indicating infection, cellular stress, or damage. Once activated, inflammasomes promote the release of pro-inflammatory cytokines, particularly interleukin-1 beta (IL-1β), and facilitate a type of programmed cell death called pyroptosis. Activating inflammasomes is a crucial part of the innate immune system’s defense mechanism, aiding in the clearance of pathogens and regulating inflammation [3].

Persistent inflammation in the liver, often resulting from various causes such as viral infections or chronic alcohol consumption, can lead to a condition known as fibrosis. This is characterized by the excessive accumulation of extracellular matrix, which can progressively disrupt the normal tissue architecture and impair liver function. If left untreated, fibrosis may advance to cirrhosis, a severe and irreversible scarring of the liver tissue, posing a significant risk to overall health. Understanding the interplay between inflammation and fibrosis is crucial for developing interventions to prevent disease progression [4].

Targeting inflammatory pathways has become a focus of therapeutic strategies for liver diseases. Anti-inflammatory agents and drugs modulating immune responses are being investigated for their potential in managing hepatic inflammation [5].

In summary, hepatic inflammation is a multifaceted process influenced by various factors, and its understanding is critical for developing targeted therapies to mitigate liver diseases. Ongoing research continues to uncover the intricate details of hepatic inflammation, providing insights into potential therapeutic interventions.

This review presents the results of studies retrieved from databases including PubMed, Scopus, the Web of Science, and Google Scholar, using the following search terms: curcumin, liver disease, liver inflammation, IFALD, NAFLD, MASLD, and PNALD.

## 2. Curcumin

**Figure 1:**
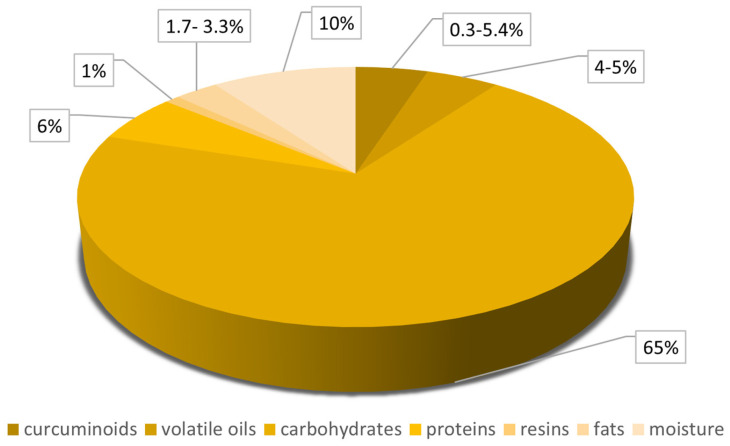
The composition of *Curcuma longa* [7].

Turmeric (*Curcuma longa*) is a plant from the ginger family (*Zingiberaceae*) that originated from India. It is used as an essential ingredient from a spice called curry, which is known around the globe for its properties like flavor and color [6]. Due to its specific yellow color, it has been used for centuries as a natural coloring agent in food (mustard sauce, chips, butter), drinks, cosmetics, and textiles. However, the most essential attributes are related to its multiple health benefits. The composition of *Curcuma longa* is shown in Figure 1.

**Figure 2:**
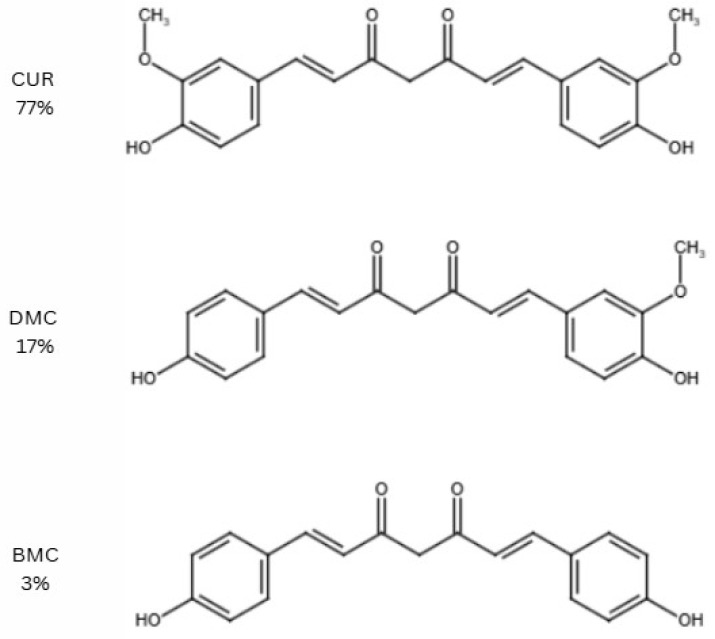
The chemical structures and percentage quantities of curcuminoids derived from *Curcuma longa* [6]. CUR: curcumin, DMC: demethoxycurcumin, and BMC: bisdemethoxycurcumin.

The group of curcuminoids, which constitutes between 0.3% and 5.4% of *Curcuma longa*, includes CUR, demethoxycurcumin (DMC), and bisdemethoxycurcumin (BMC). Figure 2 illustrates the chemical structures and the relative quantities of these curcuminoids in *Curcuma longa* [6], with CUR being the most abundant compound.

**Figure 3:**
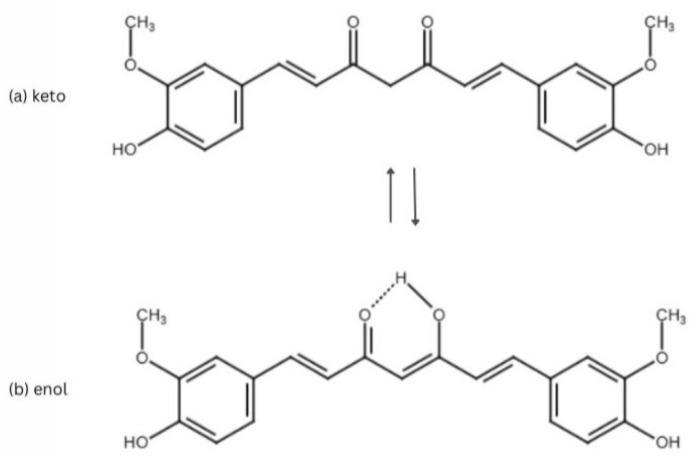
Two tautomeric forms of CUR: (**a**) keto form and (**b**) enol form.

CUR’s chemically named 1,7-bis(4-hydroxy-3-methoxyphenyl)-1,6-heptadiene-3,5-dione is classified as a polyphenol. It possesses three reactive sites: a hydrogen atom donor, a Michael acceptor, and a metal chelator. The ability to chelate the metals and form complexes with ions such as aluminum, copper, chromium, and mercury is assigned to the α,β-unsaturated β-diketone part of CUR. The β-diketo moiety of CUR occupies the role of Michael acceptor in nucleophilic addition reactions with chemical moieties such as -OH, -SH, and selenol groups [7]. Due to its lipophilic nature, CUR is practically insoluble in water at room temperature but dissolves readily in organic solvents. Despite dicarboxylic acid and polar functional groups in its central moiety, CUR’s oil–water partition coefficient (logP) ranges between 2.3 and 2.6 [8]. CUR exhibits poor stability at 37 °C in a 0.1 M phosphate buffer with neutral pH. Under these conditions, which mimic the in vivo environment, its half-life time (t_1/2_) is less than 10 min [9]. For comparison, at room temperature in 0.1 M citrate-phosphate buffer and neutral pH, t_1/2_ occurred around 20 min [10]. The keto form predominates at neutral and acidic pH, while the less stable enol form emerges at alkaline pH (Figure 3) [7,11]. The instability associated with the enol form at alkaline pH poses an additional challenge to the therapeutic potential of this substance.

The low biodistribution of CUR is primarily attributed to its extensive hepatic metabolism and the pronounced first-pass effect in the body [12]. CUR undergoes metabolism primarily through the action of NADPH-dependent reductase and alcohol dehydrogenase enzymes [12,13]. The predominant metabolites formed include tetra- and hexahydrocurcumin [12]. An alternative metabolic pathway for CUR involves its conjugation with glucuronic acid and sulfates [11,12]. Intravenous administration addresses many challenges associated with the oral delivery of CUR. The intravenous route circumvents the process of drug absorption by directly delivering the compound into the bloodstream. This approach bypasses the need for drug dissolution in body fluids, a critical limitation for poorly soluble compounds like CUR [14]. However, delivering free CUR intravenously still has limitations. It does not ensure targeted delivery to specific tissues, and the compound’s circulation time in the bloodstream remains relatively short, reducing its therapeutic efficacy [15]. Clinical trials conducted on both humans [16] and animals [17] have demonstrated that CUR exhibits inadequate absorption from the gastrointestinal tract and low bioavailability [18]. The intravenous administration of the compound also has an unsatisfactory clearance and volume of distribution [19]. The free CUR release profile is rapid, which causes an uneven delivery of the drug dose over time. Gong et al. (2021) evaluated the in vitro release of CUR using the dynamic dialysis method, showing that 60% of the polyphenol was released from dimethyl sulfoxide (DMSO) within the first 8 h of administration [20]. Due to its unfavorable pharmacokinetics and limited bioavailability, various approaches have been tested to improve CUR’s properties, including structural modifications or incorporation into nanocarriers [21].

## 3. Biological Activity of Curcumin

CUR is recognized for its multidirectional biological activity, modulating various biochemical signaling pathways and exhibiting potent antioxidant, anti-inflammatory, antimicrobial, and immunomodulatory properties. The molecule has intrinsic antioxidant capabilities, contributing to its ability to mitigate oxidative stress [22,23]. Additionally, CUR affects numerous biological targets (Table 1), allowing it to influence the pathophysiology of several diseases. These include various cancers, neurological disorders, cardiovascular and pulmonary diseases, diabetes, polycystic ovary syndrome, atherosclerosis, and metabolic syndrome [24,25,26,27].

This review focuses on the emerging evidence supporting CUR as a viable therapeutic candidate, particularly in managing and treating liver diseases, given its ability to modulate pathways implicated in hepatoprotection and liver regeneration. Its most important property is anti-inflammatory activity, manifesting in the ability to inhibit various inflammatory mediators, such as cytokines, chemokines, and enzymes like cyclooxygenase (COX) and lipoxygenase (LOX) [24]. By interfering with these pathways, CUR demonstrates its efficacy in reducing inflammation at the molecular level.

CUR is known to reduce the effects of liver damage caused by poisoning. The compound can lower the level of pathogenic biomarkers and reduce oxidative stress caused by toxins [30]. The effect of CUR on lowering the level of pro-inflammatory cytokines, diminishing the production of reactive oxygen species (ROS), and their uptake has been documented. Additionally, CUR affects cytochrome translocation, preventing mitochondrial damage [30,31,32]. The compound also affects the expression of the heme oxygenase 1 (HO-1) enzyme by acting on nuclear factor erythroid 2-related factor 2 (Nrf2), causing an antioxidant effect [30,33,34]. Moreover, in a study by Jin et al., curcumin significantly alleviated the induced liver damage caused by the environmental pollutant polybrominated biphenyl by acting on the Nrf2 pathway [35]. CUR has been shown to alleviate liver damage caused by heavy metals, such as lead, iron, aluminum, and mercury [30,33,34,36], and drugs, such as isoniazid, rifampicin, ethambutol, and pyrazinamide [37]. In cases of iron poisoning, the administration of CUR significantly reduced the levels of alanine aminotransferase (ALAT) and aspartate aminotransferase (AST), both of which serve as key indicator enzymes for liver function [7]. The administration of CUR to patients in clinical trials resulted in a decrease in the levels of liver transaminases and bilirubin. The beneficial effect may be attributed to CUR’s ability to inhibit superoxide production by macrophages, suppress inflammatory factors, modulate NF-κB signaling, and reduce lipid peroxidation. Its capacity to regulate NF-κB signaling plays a crucial role in its anti-inflammatory properties, making CUR potentially effective in managing inflammatory conditions [25,38,39]. Moreover, CUR mitigates the intensity of hepatic inflammation in an experimental model of steatohepatitis induced by the methionine and choline-deficient diet, and this impact is likely achieved through the inhibition of NF-κB activation and the corresponding pro-inflammatory genes [40]. In a recent randomized, double-blind study including 52 patients with metabolic steatohepatitis (MASH) after phospholipid CUR administration at a dosage of 2 g/day for 72 weeks, 62% of patients had MASH resolution, 50% had fibrosis improvement by ≥1 stage, and 42% had ≥2 stage fibrosis improvement, possibly through NF-kB inhibition [41].

Moreover, the hepatoprotective effect of CUR is also used in reducing adverse health effects in people addicted to alcohol. Incorporating this polyphenol into the nutritional intervention for such patients enhances liver function markers. The anti-inflammatory and antioxidant effects resulted not only in the reduction of ALAT and AST but also in gamma-glutamyl transferase (GGT) [42]. The mitigation of the harmful effects of alcohol consumption was achieved through an increase in endogenous antioxidants, including superoxide dismutase, glutathione, and glutathione peroxidase, coupled with a reduction in pro-inflammatory markers such as interleukin 6 (IL-6) and C-reactive protein (CRP) [43]. A study by Petagine et al. (2025) also showed a beneficial effect of curcumin in alcohol intoxication [44]. Pretreatment with CUR leads to increased cell viability in hepatoma cells induced by ethanol exposure and reduced ROS levels [44].

The oral use of CUR also improves liver tissue associated with fibrosis, steatosis, and cirrhosis [45]. Liver cirrhosis is a complex condition characterized by progressive tissue damage and fibrosis. While its histopathological changes are irreversible, mitigating the disease’s effects can improve patient survival. The administration of CUR has been shown to significantly decrease disease activity and severity by suppressing inflammation, oxidative stress, and angiogenesis. CUR treatment at a dose of 1000 mg/day over three months effectively reduces levels of ALP (alkaline phosphatase), bilirubin, and prothrombin time [46]. Moreover, besides biochemical parameter changes, it positively impacts the quality of life. HRQoL (health-related quality of life) is a measure that assesses physical well-being, mental well-being, and changes in functioning in society. CUR improves emotional functions in patients with liver cirrhosis by reducing the level of fear associated with the disease and depression. It had a positive effect on reducing fatigue and pain and enhancing sexual activity—enabling patients to function better in society [47].

The anti-cancer abilities of CUR have been investigated for a long time. CUR exerts anti-cancer effects by targeting growth factors, cytokines, genes, and transcription factors that regulate cell growth and apoptosis [48]. Due to its anti-inflammatory mechanism of action, CUR may appear as part of anti-cancer treatment in, e.g., pancreatic cancer [49,50], head and neck cancer [51], lung cancer [52,53], thyroid cancer [54], or liver cancer [55]. Moreover, alleviating liver fibrosis also reduces the risk of developing cancer [46]. The liver fibrosis induces apoptosis, proliferation, the migration of cancer cells, and their invasion into other tissue areas. The antioxidant nature of the compound also, in this case, has a positive effect on inhibiting the carcinogenesis process by reducing the level of ROS [56,57]. In clinical trials, in patients with locally advanced or metastatic cancer, a dose of 300 mg/m^2^ CUR did not cause significant changes in tumor size, which may suggest that CUR should be combined with other chemotherapeutics at such an advanced stage of the disease. However, its inhibitory effect on sphingosine kinase may reduce the frequency of tumor recurrence. The limited efficacy of curcumin in patients with locally advanced or metastatic cancer may be partly attributable to its poor bioavailability, and it is possible that its ability to kill cancer stem cells did not translate into tumor shrinkage due to the limited treatment duration and impaired immunological function in these patients. CUR’s circulation time in the bloodstream remains relatively short, reducing its therapeutic efficacy and resulting in poor bioavailability, which is determined by its redistribution and extensive metabolism by glucuronidation [58]. Moreover, curcumin has been shown to benefit cancer therapy by inhibiting the development and migration of tumors through epigenetic mechanisms, indicating the role of the compound as an epigenetic modulator in building cancer resistance [59]. Recent findings indicate that CUR promotes cell death in cancer models triggered by iron-dependent lipid peroxidation. The administration of the compound caused a significant increase in iron ions and a decrease in intracellular GSH levels by acting on long-chain acyl coenzyme synthetase-4 [60].

The positive effect on fatty liver disease may be exerted by reducing the level of triglycerides (TG) and cholesterol [61,62]. However, studies conducted on obese patients at a dose of 200 mg/day for 6 weeks showed no effect of CUR on lowering liver fat [63]. Despite this fact, this polyphenol can have a preventive impact on fat accumulation. In clinical studies, a reduction in anticipated food consumption was observed, contributing to lower body weight [62,63]. The mechanism is attributed to the increased secretion of glucagon-like peptide-1 (GLP-2) and peptide tyrosine-tyrosine, which are enzymes that decrease appetite [64]. Furthermore, CUR has been shown to modulate adiponectin and leptin levels, which play critical roles in regulating fat storage [65]. Notably, using Livogen Plus^®^ (Tishcon Corporation, New York, USA), a nutraceutical formulation containing CUR, significantly lowered the controlled attenuation parameter (CAP) score—a marker of liver fat deposition. The most pronounced effects were observed in patients with low high-density lipoprotein cholesterol (HDL-C) levels or those experiencing the most significant reduction in AST [66].

The study conducted by Afshar Ghahremani et al. (2024) demonstrated that CUR at 20 mg/kg/day decreases inflammatory factors such as IL-6 and tumor necrosis factor (TNF-α) and c-Fas on postnatal rats after ketamine anesthesia [67]. This study corroborated a study conducted by Abo-Salem et al. (2014) in which CUR at 200 mg/kg/day decreased IL-6 and TNF-α in diabetic rats with heart failure [68]. Guo et al. (2018) demonstrated that the oral administration of CUR at a dose of 300 mg/kg/day for 16 weeks in rats with experimental diabetes effectively reduced the production of transforming growth factor-beta1 (TGF-β1) [69]. It also suppressed TGF-β type II receptor (TβR II) levels and Smad2/3 phosphorylation while increasing Smad7 expression. Smad7 responds to TGF-β signaling by inhibiting the activity of other Smad proteins involved in signal transduction. This regulation helps maintain cellular homeostasis by preventing excessive inflammation and overactive regenerative processes [69]. Those outcomes have been confirmed in another study conducted by Ma et al. (2025) where CUR and curcumin-derived compound J7 exhibits hepatoprotective properties in a rat model of type 2 diabetes by attenuating fibrotic processes and preserving hepatic function [70]. These therapeutic outcomes appear to be linked to the suppression of the TGF-β/Smad pathway and the regulation of key apoptosis- and inflammation-related proteins, including NF-κB, BCL-2, and BAX [70]. These findings suggest that CUR can be utilized for its potent anti-inflammatory effects, as it explicitly targets key molecules involved in the inflammatory pathways.

Given the above biological properties of CUR and the evidence supporting its potential applications in the treatment of certain liver diseases, it can be concluded that CUR warrants further investigation as a therapeutic option for other liver diseases, particularly those for which effective treatments are currently unavailable and that are prevalent in the general population. This is especially relevant considering the high incidence of such diseases as MASLD and IFALD. These two liver diseases have distinct etiologies—MASLD is primarily linked to metabolic factors, while IFALD is associated with the prolonged use of parenteral nutrition (PN). However, they share similarities in their impact on liver function and disease progression. Although there is a limited number of scientific papers exploring the role of CUR in the treatment of IFALD, the presence of such studies for MASLD raises the important question: could CUR also be effective in treating IFALD? This condition is significantly more severe, as it typically occurs in patients reliant on PN, which is often their only source of nourishment and cannot be discontinued. In this article, we aim to provide a comprehensive overview of the etiology of IFALD to establish a foundation for understanding how findings from studies on MASLD patients could potentially be applied to IFALD.

## 4. IFALD

In 1981, Fleming and Remington were the first scientists to define intestinal failure (IF) as “a reduction in the functioning gut mass below the minimal amount necessary for adequate digestion and absorption of food” [71]. IF can be categorized into three types based on the duration of PN necessity and the potential reversibility of the underlying pathology: acute condition, prolonged acute condition, and chronic condition (Table 2) [72,73].

**Figure 4:**
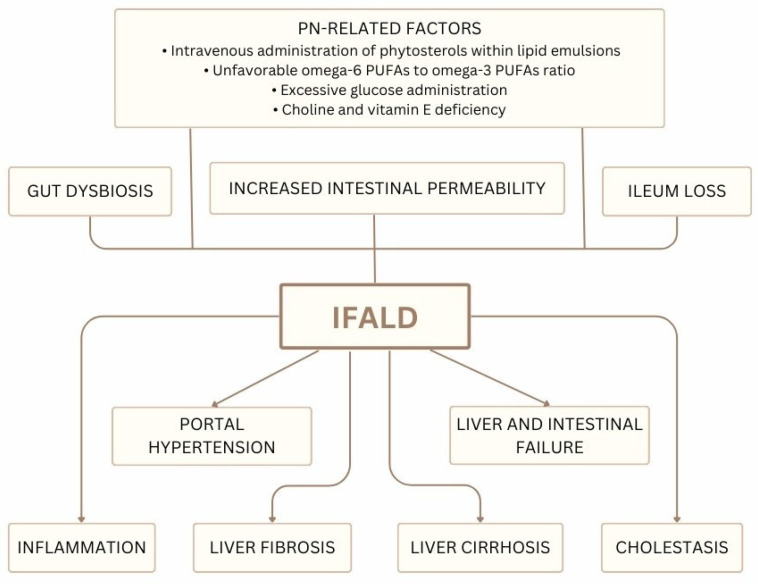
Causes and complications of IFALD [73,76,77]. PN-related factors: parenteral nutrition-related factors, omega-6 PUFAs: omega-6 polyunsaturated fatty acids, and omega-3 PUFAS: omega-3 polyunsaturated fatty acids.

PN is a medical intervention that delivers vital components like amino acids, lipids, glucose, and micro-elements to the organism without involving the stomach and intestines in digestion. In this method of clinical nutrition, those components are delivered right into the bloodstream [73]. Complications of PN can be divided into three groups: mechanical (e.g., damage to adjacent organs during line placement, hemorrhage, line displacement or line blockage, and thromboembolism), infectious (e.g., central line-associated bloodstream infections), and metabolic (e.g., hyperglycemia, metabolic bone disease, cholestasis, and transient rise in transaminases or alkaline phosphatase) [74]. One of the most severe infectious complications of PN is IFALD. It is a condition characterized by liver injury and dysfunction that occurs in individuals with intestinal failure who require long-term PN support [75]. The pathogenesis of IFALD is multifactorial and involves various factors, including congenital or acquired ileum loss, increased intestinal permeability, gut dysbiosis, and risk factors associated with PN. A summary of the causes and complications associated with IFALD is presented in Figure 4.

### 4.1. Ileum Loss

The loss of the ileum, whether partial or total, can significantly impact liver function. In IFALD patients, compared to healthy subjects, there is significantly higher cholesterol-7a-hydroxylase (CYP7A1) protein expression and lower farnesoid X receptor (FXR) expression. Moreover, lower fibroblast growth factor 19 (FGF19) levels are observed in those patients, suggesting that ileum loss or dysfunction affects the homeostasis of the bile acids [78,79,80].

The ileum plays a crucial role in the enterohepatic circulation, where bile acids are reabsorbed after their role in digestion. When the ileum is lost or damaged (due to surgical resection or disease), this reabsorption process is disrupted, reducing bile acid recycling. FGF19 is produced in the ileum in response to bile acids. This hormone plays a key role in regulating bile acid synthesis in the liver by suppressing the CYP7A1 enzyme. The loss of ileum reduces FGF19 levels and, consequently, results in a decreased inhibition of CYP7A1, leading to the overproduction of bile acids and a buildup of their toxic effect in the liver.

### 4.2. Gut Dysbiosis

Dysbiosis is characterized by decreased beneficial bacteria and an overgrowth of potentially harmful bacteria [81]. Patients receiving prolonged PN are more prone to intestinal dysbiosis due to a lack of nutrient provision to the gastrointestinal tract. The disrupted equilibrium of beneficial (*Clostridium*) and harmful (*Proteobacteria* and *Actinobacteria*) bacteria in the intestines can contribute to the progression and severity of liver diseases. This imbalance may lead to increased intestinal barrier permeability, allowing harmful substances, like lipopolysaccharides (LPS), to enter the bloodstream and exacerbate liver inflammation. Changes in the gut microbiota can promote intestinal inflammation, leading to impaired bile acid metabolism and, as a result, cholestasis [76,82]. It has been shown that patients with small intestine bacterial overgrowth (SIBO) are more vulnerable to small intestine inflammation. SIBO increases intestinal permeability, endotoxin production, and the release of proinflammatory cytokines in the liver, which can damage this organ [83]. Korpela et al. (2017) showed an association between intestinal microbiota (a high amount of *Lactobacilli*, *Proteobacteria*, and *Actinobacteria* and a small amount of *Clostridium* clusters III, IV, and XIVa), liver steatosis, and fibrosis in IF patients [84]. Understanding and addressing dysbiosis in the context of liver inflammation is crucial for developing effective therapeutic strategies to manage and potentially prevent the progression of liver disorders.

### 4.3. Intravenous Administration of Phytosterols

The key components of PN include amino acids, glucose, and lipids, which are administered in the form of an intravenous lipid emulsion (ILE). Currently available ILEs consist of various mixtures of soybean oil, olive oil, fish oil, and/or medium-chain triglycerides (MCTs) [85]. It is believed that the administration of plant oil-based ILEs, especially soybean oil, can contribute to the development of IFALD. Such ILEs are rich in phytosterols, which are structurally similar to cholesterol but act differently in the human body. Phytosterols, especially stigmasterol, are antagonists of FXR [86,87]. FXR acts as a key transcriptional regulator, influencing the expression of various genes involved in bile acid homeostasis. By activating FXR, the body can control the levels of bile acids, preventing their accumulation and potential toxicity. FXR also contributes to maintaining lipid and glucose homeostasis, making it a significant player in metabolic processes. The elevated levels of phytosterols in the bloodstream and the resulting accumulation of these compounds in the liver in patients fed parenterally, disrupting bile acid synthesis, contribute to the development of cholestasis [88]. Moreover, Ghosh et al. (2021) showed that the combination of sitosterol and stigmasterol co-incubated with a liver receptor homolog-1 (LRH-1) inverse agonist was shown to exacerbate liver dysfunction by reducing the expression of bile acid transporter genes, such as multi-drug resistance protein 2 (ABCC2/MRP2), small heterodimer partner (SHP), and bile salt export pump (BSEP) [89]. Additionally, the same authors have demonstrated that phytosterols can suppress ATP-binding cassette transporters G5 and G8 (ABCG5/8), the primary neutral sterol transporter responsible for hepatobiliary and transintestinal phytosterols and cholesterol excretion, through mechanisms such as RNA interference or liver X receptor (LXR) antagonism. This suppression leads to the accumulation of phytosterols in the liver, further disrupting FXR binding to key promoters and exacerbating cholestasis [89]. The results of clinical studies confirmed the importance of phytosterols in the pathogenesis of IFALD. Mutanen et al. (2014) have shown a connection between serum phytosterol levels and liver injury in IFALD [77]. Reducing the amount of solely soybean oil-based ILE from 2–3 to 1 g/kg/day [90] or substituting it with fish oil-based ILE at 1 g/kg/day or with ILEs based on a mixture of several oils, including fish oil at the maximum dose of 2–3 g/kg/day [91] are experimentally proven methods that can reverse cholestasis in infants with IFALD. Studies on mouse models have proven that an increased absorption of LPS from the injured intestine, and a high supply of phytosterols with PN can lead to IFALD [88,92].

### 4.4. Other PN-Associated Risk Factors

Several commercial intravenous lipid emulsions (ILEs) that differ in oil phase composition and fatty acid profile are available. Their composition is recognized as another key factor contributing to the development of IFALD. A high concentration of omega-6 polyunsaturated fatty acids (PUFAs) and a low concentration of omega-3 PUFAs in soybean oil-based ILE is unfavorable. Such a composition of PUFAs can generate pro-inflammatory eicosanoids and leukotrienes [73,75], leading to inflammation, which, over time, may exacerbate PN-associated liver complications. Therefore, the proper ratio of omega-6 PUFAs to omega-3 PUFAs (ranging between 1:1 and 4:1) should be maintained to protect the liver.

Another important factor associated with the pathogenesis of IFLAD is the provision of glucose. Its infusion at rates >5 mg/kg/min [93] and elevated molar ratio of portal venous insulin to glucagon due to the PN administration with high glucose concentration [94] seem to induce hepatic steatosis. A prophylactic seems to be crucial for the prevention of IFALD during PN therapy. Research has demonstrated that PN cycles ranging from 8 to 12 h are more favorable than a continuous model of PN administration, which lasts 24 h/day, due to the reduced risk of hepatic injury and the potential to stabilize liver function [95].

It has also been proven that vitamin E deficiencies are associated with developing liver steatosis [96]. Tocopherol (vitamin E) is a significant antioxidant that protects cells from oxidative stress. Due to its unique characteristics, it is incorporated into intravenous emulsions as an excipient and added to PN as a component of vitamin preparation. In a study conducted by Ng et al. (2016) involving PN administration to preterm piglets, it was demonstrated that in contrast to bare Intralipid administration, the addition of tocopherol at a dosage of 251 mg/L to Intralipid or the provision of tocopherol presented in Omegaven effectively increased biliary and lipidemic markers associated with IFLAD in serum and liver tissues [97].

Moreover, choline deficiency may also play a role in the development of IFALD. Buchman et al. (2001) provided information suggesting that choline deficiency causes hepatic abnormalities in adults [98], and Sentongo et al. (2010) confirmed it in infants [99]. Buchman et al. (2001) proved that over 24 weeks of choline supplementation significantly improved hepatic enzyme parameters [98]. Adding choline to PN shows promise in preventing IFALD by reducing oxidative stress, improving hepatic fat export, and promoting the catabolism of fatty acids in PN-fed immature rats by enhancing choline and phosphocholine levels and reducing betaine liver levels. Additionally, choline supplementation reversed peroxisomal proliferator-activated receptor alpha (PPARα) promoter hypermethylation, upregulated PPARα, carnitine palmitoyltransferase 1 (CPT1) mRNA and protein expression, compared to rats receiving PN alone [100].

### 4.5. IFALD: Diagnosis and Treatment

Despite intensive research, IFALD diagnosis is challenging. Table 3 shows some of the noninvasive diagnostic biomarkers, imaging techniques, and physical features of IFALD.

A diagnosis should never be made based on a single diagnostic parameter. Conducting a series of tests is recommended to confirm or exclude the likelihood of IFALD occurrence. The most important parameters that should be considered during diagnosis are liver enzyme levels, bilirubin concentrations, changes in lipid profiles, and the evaluation of liver condition through ultrasound, which can provide information on steatosis or fibrosis [108]. When noninvasive techniques fail to provide sufficient information for a correct diagnosis, a liver biopsy is considered the final diagnostic procedure; at the same time, it represents one of the most invasive yet effective methods of diagnosing. A systematic review by Lee et al. (2020) revealed that the overall incidence of cholestasis is 29.9% [75]. The highest incidence, 49.8%, was observed in children with IFALD, and it can rise to as high as 90% in preterm infants receiving parenteral nutrition for more than 90 days [75].

IFALD significantly impacts both the quality and duration of patients’ lives. Unfortunately, treatment options remain limited, with ursodeoxycholic acid (UDCA) being one of the primary therapies. UDCA acts as a competitive inhibitor of the ileal absorption of harmful endogenous bile salts while demonstrating cytoprotective, antiapoptotic, immunomodulatory, and antioxidant properties in the liver [109]. It has been shown that in adults, an oral dosage of 11.2 ± 0.8 mg/kg per day reduces increased liver enzymes and direct bilirubin levels [109,110]. Moreover, UDCA induces the remission of cholestasis in children, as proven by studies conducted by De Marco et al. (2006) [111]. After the oral administration of UDCA at 30 mg/kg/day for 6 months, serum levels of cholestasis markers in children with IFLAD were significantly lower [111]. Other studies showed that GLP-2 demonstrated a good effect on the IFALD course, with improvements in hepatic steatosis in a rat model [112] or hepatic excretory function [113]. However, in phase 2 clinical trials, the increased liver stiffness after such treatment was revealed [113]. Studies performed by Fligor et al. (2023) on preterm piglets with IFALD treated with SEFA-6179 (a first-in-class GPR84/PPARa/PPARg agonist) showed that the orogastric administration of SEFA-6179 at the dose of 48 mg/kg/d for 14 days along with PN therapy prevented cholestasis and steatosis [114]. It also reduced bile duct proliferation and fibrosis [114]. In another study performed by Fligor et al. (2024), potential therapeutics, including infliximab, methylprednisolone succinate, simvastatin, and obeticholic acid were identified as predicted upstream master regulators that may reverse the PN-induced gene dysregulation [108].

Otherwise, IFALD patients may necessitate intestinal transplantation, liver transplantation, or a combined liver and intestinal transplantation [115]. Qualifying the patients for transplantation is challenging due to the unclear indications and the absence of sharp boundaries between successive phases of the disease. Isolated intestinal transplantation may be considered in patients who suffer mild or moderate IFALD [116]. Portal hypertension, fibrosis, or cirrhosis are indications for a combined liver and intestine transplant [115]. Among transplant patients, depending on their age and type of transplant, there is a significant difference in the survival ratio. According to the OPTN/SRTR 2022 Annual Data Report, patient survival rates for the 2015–2017 group, categorized by whether they received the intestine transplant or the liver and intestine transplant, pediatric patients who underwent intestinal-only transplants had better survival rates than adults over 5 years (77.2% vs. 61.3%). The survival rates for the same cohort are slightly lower for the recipients of an intestine with liver transplant. Pediatric patients had higher survival rates (66.7%) than those who underwent the transplant as adults (46.7%). Patient survival rates for the 2015–2017 group, categorized by whether they received the intestine allotransplant or the liver and intestine allotransplant, demonstrate higher short-term survival for recipients of intestine-only transplants (82.1% survival at 1 year for isolated intestine recipients compared to 62.2% for combined intestine and liver recipients). However, this disparity gradually diminished over time, with 47.6% survival at 5 years for isolated intestine recipients and 42.2% for recipients of combined intestine and liver transplants [117].

Despite identifying several promising pharmacological candidates, effective treatments or preventive strategies for IFALD are still unavailable. Consequently, research is focused on recognizing alternative bioactive compounds with anti-inflammatory properties that may enhance hepatic function in PN patients. One such promising compound is CUR. Studies in both humans and animals have demonstrated that CUR can be safely used even in high doses, which supports its potential therapeutic use, particularly for conditions like MASLD and IFALD, where no effective registered treatments currently exist [118].

## 5. MASLD

**Figure 5:**
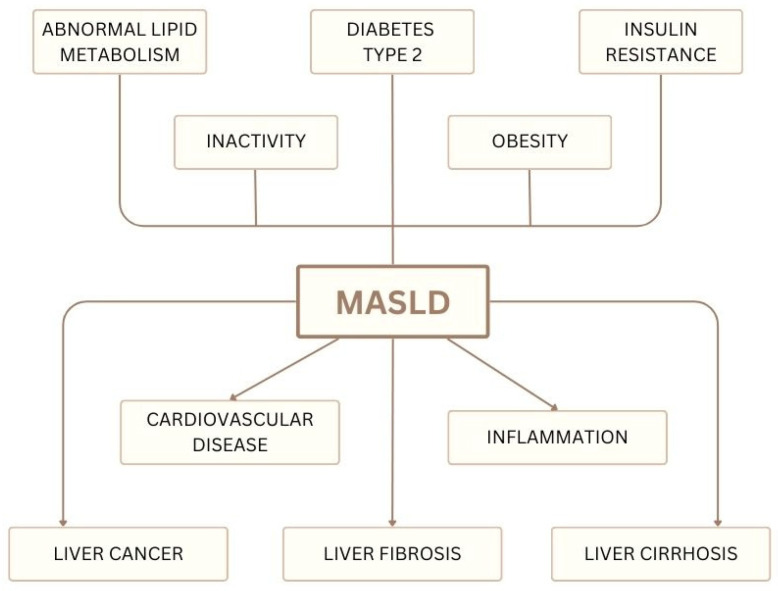
Causes and complications of MASLD [45,66,122].

Currently, one of the most common liver diseases in the world, which causes liver cancer, fibrosis, and cirrhosis, is MASLD [63]. MASLD is strictly linked to metabolic abnormalities, and to emphasize that, a group of international experts proposed in 2020 to rename the first name non-alcoholic fatty liver disease (NAFLD) as metabolic dysfunction-associated fatty liver disease (MAFLD) [119]. The concept of MAFLD represents a shift from the exclusionary term non-alcoholic, emphasizing that metabolic dysfunction is central to disease development while also recognizing that alcohol intake or viral infections may contribute to its progression. In line with this updated understanding, international hepatology societies have proposed the use of MASLD for metabolic dysfunction-associated steatotic liver disease and metabolic steatohepatitis (MASH) to describe its inflammatory form, replacing the term non-alcoholic steatohepatitis (NASH) [120,121]. It most often occurs in people with type 2 diabetes and diagnosed obesity. Additionally, in the event of the above-mentioned disturbances in the functioning of the body, cardiovascular diseases often occur, which are also the primary cause of death in patients diagnosed with MASLD [66,122,123]. A summary of the causes and complications associated with MASLD is presented in Figure 5.

There are two fundamental theories in the literature regarding the pathogenesis of MASLD, called the “two-hit theory”. The first possibility refers to the development of MASLD primarily caused by insulin resistance. According to this theory, insulin resistance contributes to the excessive accumulation of free fatty acids in the liver, which is caused by a reduced process of oxidation. As a result of this mechanism, liver steatosis occurs. The second theory assumes that excessive fat deposition in liver cells is the primary source of MASLD. This causes inflammation from oxidative stress, mitochondrial dysfunction, and lipid peroxidation. Consequently, it causes liver fibrosis associated with the repair process of damaged organ cells [124].

The main factors causing MASLD are oxidative stress [125] and lipid peroxidation, mainly from TG [124]. Lipids in liver cells are accumulated as a result of abnormal mitochondrial biogenesis. It has been proven that postnatal overfeeding in rats may lead to an increased risk of MASLD in adults [126]. Moreover, a high-fat diet may modify intestinal permeability and increase the level of LPS in blood serum, causing an increased activation of pro-inflammatory pathways [127]. According to forecasts, the likelihood of MASLD in society in the coming years will become the leading cause of liver transplantation by 2025 [128].

Both in MASLD and IFALD, the anti-inflammatory action is relevant, as in both cases, the presence of hepatic steatosis can lead to liver inflammation, known as MASH, which may progress and increasingly impair the function of the organ, potentially leading to liver cirrhosis or even cancer [105,129,130,131].

## 6. Curcumin’s Potential in IFALD and MASLD Therapy

CUR has many properties that make it a potential candidate for clinical trials in MASLD and IFALD. Common criteria for patient inclusion in the study were the diagnosis of MASLD by ultrasound or Fibroscan. Exclusion criteria in the clinical trial included the following: (1) pregnancy or breastfeeding; (2) hypersensitivity to CUR; (3) alcohol consumption; (4) smoking; (5) taking medications or dietary supplements that affect the functioning of the liver; (6) kidney diseases, lung diseases, thyroid diseases, coronary diseases, renal diseases, and Wilson’s disease; (7) the occurrence of cancer; (8) weight loss in a specific period; and (9) the occurrence of MASLD secondary to alcohol consumption. In clinical trials, CUR was shown to be safe for use without causing any serious side effects [56,102,103,111,112,113,114,115,116,117,118,119,120,121,122,123].

**Figure 6:**
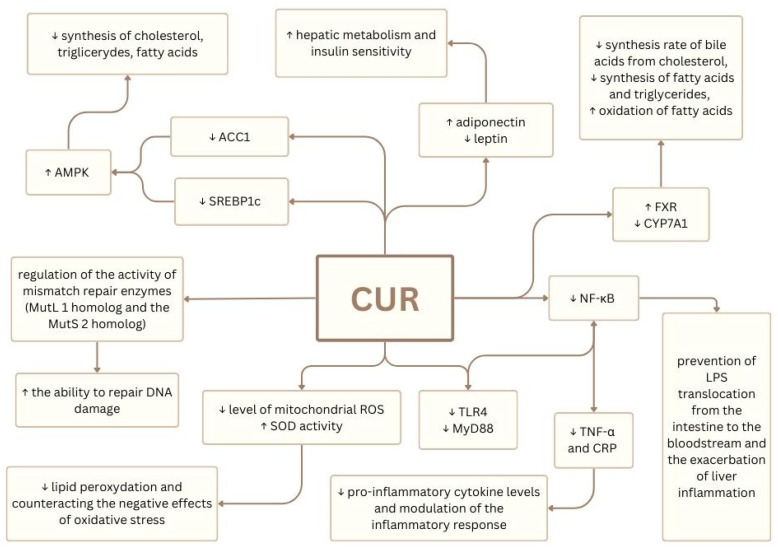
The schematic effect of CUR signaling pathway in IFALD and MASLD. CUR affects multiple key targets, including, e.g., NF-κB, AMPK, TLR4, MyD88, CRP, FXR, SOD, and ROS [57,58,65,122,126,127]. ↑ increased, ↓ decreased, AMPK: activated protein kinase, LPS: lipopolysaccharides, NF-κB: nuclear factor-kappa B, TNF-α: tumor necrosis factor, CRP: C-reactive protein, TLR4: toll-like receptor 4, MyD88: myeloid differentiation primary response 88, ROS: reactive oxygen species, SOD: superoxide dismutase, FXR: farnesoid X receptor, CYP7A1: cholesterol-7a-hydroxylase, ACC1: acetylo-CoA carboxylase, and SREBP1c: sterol regulatory element-binding protein 1c.

The molecular mechanisms summarized in Figure 6 are supported by the referenced studies illustrating CUR’s role in modulating the inflammatory and metabolic pathways in MASLD and IFALD [57,58,65,122,126,127].

The key effect of the compound is its antioxidant potential by reducing the level of mitochondrial ROS and increasing the activity of superoxide dismutase (SOD) [126,132]. In MASLD, there is an excessive accumulation of lipids in liver cells, mainly TGs. Mitochondria play a major role in lipid metabolism. They are responsible for structural modifications of lipids in the tricarboxylic acid cycle, allowing them to be packaged into lipoproteins, which are then stored as fat droplets in the body. The excessive accumulation of TGs affects mechanisms dependent on redox reactions, indirectly generating ROS [124]. Lipid peroxidation, resulting from excessive ROS production, causes the activation of stellate cells. This results in the possibility of fibrogenesis and inflammation, and in further stages, liver fibrosis and cirrhosis. Activated hepatic stellate cells (HSCs) are the primary mediators of fibrogenesis, producing excessive extracellular matrix proteins that contribute to liver scarring. Their activation is primarily driven by transforming growth factor-beta (TGF-β), which signals through Smad3 phosphorylation at the C-terminal MH2 domain.

Additionally, the MAPK signaling cascade can modify Smad3 at its linker domain, leading to increased PAI-1 production and stimulating both the proliferation and migration of HSCs. These pathways highlight critical molecular targets for developing antifibrotic therapies [28]. Several studies showed that CUR administration effectively ameliorated liver fibrosis and reduced TG levels [66,118,133,134].

The accumulation of free fatty acids is mediated by the PPARs, LXR, and activated protein kinase (AMPK). PPARs reduce oxidative stress and increase the secretion of beneficial adipocytokines and anti-inflammatory factors. It has been proven that PPAR agonists can protect against the development of MASLD [135,136]. PPAR activation leads to the expression of the Nesfastin-1 gene, which inhibits appetite and fat storage through an AMPK mechanism mediating glucose metabolism. The oral administration of nanoCUR at a dose of 80 mg/day for 3 months increased Nesfastin-1 levels. Changing the neuropeptide concentration indirectly resulted in an improvement in the lipid and glucose profiles. [137]. This effect is caused by the activation of AMPK as a result of the phosphorylation of acetyl-CoA carboxylase 1, and the binding of sterol regulatory element-binding protein 1c (SREBP-1c), which results in the inhibition of the synthesis of cholesterol, TG, and fatty acids [65]. Additionally, CUR has been reported to enhance insulin responsiveness and diminish hepatic lipid accumulation, potentially through the stimulation of the PI3K/Akt signaling cascade [138].

Another aspect of the harmful effects of ROS is the generation of an inflammatory response and the associated mobilization of the body to produce pro-inflammatory cytokines such as TNF-α and CRP [65,82,122]. In clinical trials, CUR significantly reduced TNF-α, a pro-inflammatory cytokinin, and high-sensitivity C-reactive protein (hs-CRP). An indirect effect on the NF-κB pathway in peripheral blood mononuclear cells, which is involved in the inflammation cascade, has been proven [122]. When patients were orally administered nanoCUR at a dose of 80 mg/day for 3 months, in addition to confirming the effect on the previously mentioned inflammatory factors, it also reduced the level of IL-6 [137]. In the case of the administration of a curcuminoids–piperine complex in the form of a combination of C3 Complex^®^ plus Bioperine^®^ (500 mg curcuminoids and 5 mg piperine) daily for 8 weeks, additional measurements of the levels of IL-1α, IL-1β, IL- 2, IL-4, IL-6, IL-8, IL-10, monocyte chemoattractant protein 1 (MCP-1), interferon γ (IFN-γ), epidermal growth factor (EGF), and vascular endothelial growth factor (VEGF) were taken. However, most of the above-mentioned cytokines and growth factors were not affected by treatment with the complex. Beneficial changes were observed for MCP-1, EGF, and TNFα [139]. The progression from MASLD to liver fibrosis and hepatocellular carcinoma is a common clinical trajectory. A 2024 meta-analysis by Li et al., covering 52 studies up to 2021, found that curcumin effectively slows this progression by reducing inflammation. Hepatoprotective effects were observed at doses of 100–400 mg/kg over 4 to 10 weeks through the modulation of key signaling pathways, including TLR4/NF-κB, Keap1/Nrf2, Bax/Bcl-2/Caspase-3, and TGF-β/Smad3 [140].

ROS may also contribute to damage to the DNA structure. In this case, there is a possibility of a change of cytosine to 5-hydroxyuracil or uracil due to deamination [118]. CUR also plays an important role in regulating the methylation level of mismatch repair enzymes. The hypermethylation of these compounds causes the inactivation of enzymes, which reduces their expression and, consequently, mitigates mutations in the synthesized DNA chain. CUR significantly affects the level of the methylation of the proximal and distal promoter regions of the MutL 1 homolog and the MutS 2 homolog. However, the effect on the concentration of 8-hydroxy-2′-deoxyguanosine (8-OHdG), causing guanine oxidation, is not clear in the studies. At a CUR dose of 250 mg/day, through an 8-week study conducted by Hariri M et al. (2020), no effect on 8-OHdG reduction was demonstrated [118]. In turn, Mirhafez SR demonstrated that the DNA damage marker was significantly reduced with carboxymethyllysine, affecting the generation of free radicals [118,141].

CUR in both the phytosomal form and the curcuminoid complex with or without piperine reduced the levels of cholesterol, TG, and low-density lipoprotein cholesterol (LDL-C) in patients with MASLD [121,133,137,142,143]. The effect on HDL-C was variable in clinical studies. When using 70 mg of curcuminoids in the form of an amorphous dispersion and 800 mg of phytosomal CUR (200 mg of CUR), there was a significant increase in the therapy lasting 8 weeks [133,142]. In turn, 2 months of the use of phospholipid-based phytosomal CUR at a dose of 250 mg/day reduced the level of HDL-C [144]. Phytosomal CUR in combination with soy phosphatidylcholine at a dose of 1000 mg/day for 8 weeks did not change this parameter [34]. The use of CUR in phytosomal form or in combination with piperine in clinical trials was aimed at improving the bioavailability of CUR. Combining CUR with phospholipids improves intestinal absorption and reduces the chances of the enzyme metabolizing the drug too early. Piperine reduces the elimination of CUR in urine as a result of conjugation with glucuronic acid. Moreover, the inclusion of piperine in the preparation enhances the effect of CUR due to its hepatoprotective, antioxidant, and anti-inflammatory properties [63,133,145].

CUR also reduces the expression level of genes such as fatty acid binding protein 1, apolipoprotein C-III, and glycerol kinase, which influence lipids’ metabolism and transport, including cholesterol [124]. Another mechanism influencing cholesterol metabolism is the reduction of the expression of the Nrf2 transcription factor and the FXR. The action is mediated by a reduced expression of SREBP-1c and reduced fatty acid synthesis [146]. Moreover, inducing FXR expression affects the synthesis of CYP7A1 and also limits the synthesis rate of bile acids from cholesterol. This affects lipid metabolism and reduces inflammation caused by the accumulation of bile acids in the liver, which is why CUR could be a strong drug candidate for IFALD therapy. Nrf2 and FXR function in a complementary manner to regulate oxidative stress and inflammatory responses within the liver. Nrf2 predominantly governs antioxidant defenses by neutralizing ROS, whereas FXR plays a key role in maintaining bile acid balance and mitigating bile acid-driven oxidative stress [147]. Their integrated activity is crucial for preserving hepatic homeostasis and limiting the advancement of chronic liver pathologies like MASLD or IFALD.

CUR reduces the level of succinates and citrates resulting from the disruption of the tricarboxylic acid cycle pathway in MASLD. The disruption of the presented pathway causes mitochondrial dysfunction, leading to oxidative stress [126,145]. In a clinical study using Meriva^®^ (INDENA S.p.A., Milano, Italy) at a dose corresponding to 50 mg/day of pure CUR for 8 weeks, the effect of the polyphenol on the occurrence of metabolites in the serum of patients with MASLD was examined. Compared to the placebo group, in addition to a decrease in the level of succinates, a decrease in the concentration of α-ketoglutarate and citrate was observed. The last of the mentioned metabolites is formed in the TCA cycle as a result of the conversion of free fatty acids to acetyl-CoA, which in the final stage is transformed into citrate [145].

The compound also indirectly affects insulin, reducing the symptoms of MASLD associated with the onset of type 2 diabetes. CUR may affect the levels of adiponectin and leptin in blood serum. These are compounds that influence the body’s sensitivity to insulin. When fat agglomerates in the body, leptin levels increase, and adiponectin decreases. CUR has an antagonistic effect on the response presented above, positively influencing glucose metabolism in the body despite its low bioavailability [65,122].

The level of adiponectin and leptin influences fat storage. It has also been shown that reduced sensitivity to leptin is associated with obesity and insulin resistance [65,135]. Adiponectin levels influence glucose metabolism, indicating antidiabetic effects. CUR affects insulin release and enhances glycolysis and glycogen synthesis while reducing gluconeogenesis in the liver and increasing glucose uptake by skeletal muscles [123,133]. A decrease in glycated hemoglobin (HbA1c), fasting blood sugar, and insulin was observed in patients treated with CUR [133,137,142].

CUR supplementation significantly reduced circulating levels of key peptides for insulin resistance that are strongly engaged in inflammation and apoptosis, glycogen synthase kinase-3 β (GSK-3β) (−2.4 ± 0.4 ng/mL vs. −0.3 ± 0.6, *p* = 0.0068) and islet amyloid polypeptide (IAPP) (−2.0 ± 0.7 ng/mL vs. 0.4 ± 0.6, *p* = 0.0163) levels, compared with the placebo group. Additionally, CUR has been found to activate AD-dependent deacetylase sirtuin-1 (SIRT1) involved in glucose homeostasis in the liver by deacetylating the peroxisome proliferator-activated receptor-gamma coactivator-1 alpha (PGC-1α) gene and inducing downstream target genes, such as PPARγ [148]. PPARγ shapes the immune response, especially the course of inflammatory reaction.

Moreover, a high-fat diet may increase intestinal LPSs, which constitute a group of endotoxins, and their increased levels have been observed in MASLD models in rats. LPSs constitute a group of ligands for the pattern recognition receptor of the innate immune system toll-like receptor 4 (TLR4). TLR4 interacts with adopter molecules for the myeloid differentiation primary response 88 (MyD88) gene, which influences NF-κB. The presented transcription factor is involved in producing an inflammatory reaction in the body [124]. CUR prevents the translocation of intestinal LPS by reducing intestinal permeability and affecting TLR4 and MyD88 [58,127,149]. The compound under investigation also exerts a beneficial influence on the alterations in gut microbiota induced by MASLD. The administration of CUR led to a reduction in hippurate endotoxins and a decrease in the concentration of bacterial-derived secondary bile acids in the serum [115]. The summary of the effects of different doses and formulations of CUR on the biochemical parameters and BMI occurring in MASLD, liver cirrhosis, and hepatic steatosis are shown in Table 4. Those findings may also be relevant to IFALD.

## 7. Curcumin’s Nanoformulations

CUR is a compound with multidirectional action that presents a broad spectrum of possible disease treatment applications. Regrettably, unfavorable pharmacokinetics after administration disable its use as a therapeutic agent. To benefit from the wide possibilities of CUR as an agent in multiple therapies, biodistribution should be improved [61,152].

Well-known drug delivery system (DDS) matrices for this aim are lipids [61,153,154]. The most common choices are liposomes, solid lipid nanoparticles, nanoemulsions, or lipid nanocapsules. Liposomes are characterized by appropriate biocompatibility and the possibility of the encapsulation of lipid and hydrophilic drugs. This allows CUR to potentially be delivered with another therapeutic agent to the target tissue [155,156,157]. Additionally, there is an opportunity to add polyethylene glycol to the phospholipid layer, increasing the hydrophilic properties of the formulation and slowing down the release of CUR from the phospholipid core [158]. Unfortunately, in some in vivo studies, liposomal CUR led to intravenous administration changes in the morphology of red blood cells and the occurrence of anemia or hemolysis [21,154].

Solid lipid nanoparticles (SLNs) are a promising alternative among lipid drug delivery systems. They have a large specific surface area, which is an important element for adapting the DDS to reach the target organ. They are characterized by great bioavailability and chemical stability. SLN is also the preferred formulation in planning to increase industrial production or sterilization compared to emulsions and liposomes [157]. Intravenous administration formulations based on SLN show high levels of CUR encapsulation and prolonged blood circulation time compared to CUR injection without a nanocarrier [61,157]. Another way to extend the remaining drug in plasma is by coating the nanoplatforms with specific molecules. One of the examples was mentioned previously: polyethylene glycol (PEG). This molecule increases the stability of drug formulation in blood plasma and reduces its chances of aggregation [152,159]. In a study by Chirio D et al. (2019), SLN combined with PEG modification protected against degradation in the aquatic environment [157]. After 24 h, the concentration of CUR in PBS was 94% of the initial encapsulation value. A slower release profile was also observed compared to free CUR. Maximum accumulation in organs after the injection of the compound occurred later than in the case of free CUR, suggesting an increased circulation time of the nanoformulation in the blood [157].

Another important aspect of intravenous administration is maintaining the stability of nanoformulation with CUR in the blood. Micelles made of poly(ethylene glycol)-b-poly(N-2-benzoyloxypropyl methacrylamide) retained their size and particle distribution during a 24 h incubation in human albumin serum at a concentration of 0.3 mg/mL, within sink conditions. Additionally, they showed that the circulation time of CUR in the blood was extended by approximately five times compared to its free form. These results indicate the potential for the intravenous administration of CUR in polymer-based formulations [160].

However, improving the pharmacokinetic properties does not mean producing a therapeutic effect. If the effect of CUR is desired in cancer therapy, the pH sensitivity or reduction mechanism strategy is used [20,161,162]. Cancer cells have a much higher concentration of glutathione than normal cells. Glutathione is a reduction agent that breaks down disulfide bonds. The structure of polymers allows modifications by cross-linking with disulfide between the molecules. In the case of contact with glutathione, they are torn apart, and consequently, the compound contained in their core is released. This strategy was used in telodendrimer-based polymer micelles (mPEG-PLA-(LA)_4_). The intravenous administration of micelles resulted in the permanent retention of CUR in the bloodstream without the effect of its premature release. Only after the nanoformulation was delivered to the tumor tissue did it disintegrate and increase the compound’s release, providing a targeted therapy mechanism.

Cross-linked micelles thus improve CUR half-life and mean residence time in blood, decrease clearance, and increase plasma concentration. The concentration of intravenous administration was 20 mg/kg and showed a similar effect to the oral administration of free CUR in a dose of 10 g [161]. The same mechanism has also been used in formulations based on hyaluronic acid against brain glioma. After intravenous injection, the formulation is characterized by minimal drug leakage in the blood and prolonged circulation, and upon delivery to glioma cells, rapid release occurs, leading to increased apoptosis compared to CUR solution. The good uptake in the brain was also influenced by the functionalization of Tween-80 and the size below 100 nm, thus allowing it to cross the blood–brain barrier [163]. Coating the particles with Tween 80 causes absorption on the surface of blood proteins: ApoE and ApoB result in receptor-mediated endocytosis transfer to the brain [154]. Another mechanism used in targeted action against cancer is pH responsiveness. A human heavy chain apoferritin (HFn) drug carrier with CUR was used to test the specificity of the effect on breast cancer after intravenous administration. HFn has the property of reorganizing its structure under the influence of pH. In a neutral environment, it retains its quaternary structure, maintaining the continuity of the nanoparticle. In turn, in a strongly acidic environment; characteristic of cancer tissue, its structure is broken down into individual units, releasing the drug. Additionally, HFn is recognized explicitly by the transferrin receptor, which is overexpressed in this tissue [162]. The acidic environment of cancer cells can be beneficial for the increased cytotoxicity effect of ZnO nanoparticles with CUR. In contact with lower pH, ZnO collapses to Zn^2+^, which presents a potential treatment modality for cancer therapy [161].

Polymer or lipid-based drug delivery systems are not the only common choices for testing CUR delivery. Nanostructures covered with a cell membrane show excellent biomimetic properties, reducing cytotoxicity and immunogenicity. Strategies to load the antioxidant CUR into cell membrane-coated nanoparticles were used to cross the blood–brain barrier for Alzheimer’s therapy. Albumin nanoparticles camouflaged with the red blood cell membrane effectively delivered the drug to neuronal mitochondria. The nanostructure demonstrated a long circulation time in the blood, safety, and prolonged release of CUR. Additional surface modification with T807 and triphenylphosphine (TPP) resulted in a specific effect on mitochondria contained in nerve cells. Consequently, an in vivo study conducted on mice showed significant improvement in cognitive abilities [164]. Enriched cell membrane vesicles are also selected for genetic modification. In a study by Wang et al. (2021) through the genetic engineering of cells, an enriched expression of the chemokine receptor type 4 (CXCR4) membrane receptor was introduced. It binds specifically to factor 1 derived from stromal cells, the excess of which is found in inflammatory tissues. The formulation showed targeted and anti-inflammatory effects in animal models while maintaining biocompatibility [165]. Interestingly, not only the membranes of healthy cells are tested with therapies. To increase the delivery of CUR and chlorin e6, the polymeric nanoparticles were coated with a tumor cell membrane homologous to targeted cancer cells. The coating increased cellular uptake at a tidy site and showed stronger anti-proliferation effects than bare NP-based nanostructures with active compounds. However, formulation still showed biodistribution to the kidneys and liver, which, as a result, will remove the drug from the body too early [166].

Iron-based nanostructures also proved effective in enhancing the effect of reducing inflammation in animal studies, where the metal acted as a nanoenzyme to remove inflammatory mediators—intracellular ROS. The combination provided a synergistic effect with CUR [15]. In turn, magnetite nanoparticles with CUR showed increased magnetic resonance imaging contrast and hyperthermic properties, enabling more detailed biodistribution imaging and a therapeutic effect at a dose of 120 mg/kg [167]. Mesoporous polydopamine is another combination of metal-based drug delivery systems that enhances curcumin action. This association increased the antioxidant capacity of CUR itself and the deposition of collagen fibers in liver fibrosis and provided new possibilities, such as monitoring biodistribution and enabling combination photothermal therapy [168].

This huge selection of platforms allows scientists to choose the most suitable one regarding the place of delivery and mechanism of action. The nanoencapsulation of CUR generates many benefits regarding the biodistribution and pharmacokinetics of the substance. The advantages include extending the time the drug stays in the blood circulation, reduced clearance, reduced susceptibility to degradation, and increased bioavailability. However, only a few tested nano-sized formulations on animals had results enabling them to be used for clinical trials [153].

## 8. Biocompatibility of Curcumin’s Nanoformulations

The mere improvement of CUR’s solubility and cellular uptake is not an indicator for applying the formulation. Drug delivery systems should be examined for their safety. In the case of the intravenous administration of the nanoformulation, the hemolytic potential, toxicity to various tissues in vivo, anthropometric measurements, and behavioral changes were assessed [21,155,167]. The highest biocompatibility among animal studies characterized nanoemulsions and formulations based on the outer membrane of cell origin. The tested CUR nanoemulsions did not negatively affect red blood cells and did not cause their breakdown. However, changes in MRC5 cell viability were observed when benzyl alcohol was used to prepare nanoemulsions. For this reason, the use of some solvents in the preparation should be avoided to eliminate cytotoxic effects [8,14]. The recent study performed by Czerniel et al. (2025), where four nanoemulsions with curcumin were developed, showed no hemolysis in any of those nanoformulations, and three of them exhibited better cell viability at low concentrations (up to approximately 4%) compared with commercial formulations [169].

The high safety of use has been demonstrated by structures composed of naturally occurring compounds or cell membranes from the body. The lack of an introduction of foreign compounds into the body minimizes immunogenicity and increases the potential biocompatibility of the entire formulation [162,164]. Hollow cage human apoferritin drug carriers for CUR showed the given characteristics. Additionally, in studies on mice, no body weight or pathological changes were observed in the heart, liver, spleen, lungs, and kidneys. The levels of ALAT, AST, and blood urea nitrogen (BUN) biomarkers for the liver and kidneys were normal. So were red blood cells, white blood cells, and platelets levels [162]. Structures made of naturally occurring compounds such as fibroin and alginate also showed no toxicity. Fibroin-based nanoparticles were not cytotoxic for Raw 264.7 macrophages or red blood cells. Moreover, during in vivo studies, no histopathological changes or potential nephrotoxicity or hepatotoxicity were observed [170]. Both preparations did not cause changes in the weight of animals, and in the case of alginate nanoparticles, no increase in DNA damage in bone marrow cells was demonstrated [21,170]. The use of cell membranes reduces immunorecognition and therefore, the possibility of causing inflammation. CUR formulations use red blood cell, tumor, and MC-3TC cell membranes for this purpose [164,165,166]. In the case of the last vesicle, the selection of this line was also dictated by the favorable properties of this cell line. It exhibits a short mitotic cycle, immortalized lifespan, and ease of transfection by lentivirus; enabling the introduction of genetic modifications to produce desired molecules in a targeted manner. A significantly lower distribution in kidney and liver tissues was also demonstrated compared to using only nanoparticles without a cell coating [165].

Liposomes were the exception to the correlation between building materials derived from natural compounds and low toxicity. The Lipocurc™ formulation had a hemolytic effect. Abnormalities in red blood cell count were observed, but no such changes were noticed with intrapleural administration. Intravenous administration at a dose of 16 mg/kg in rats resulted in numerous spiny protrusions in the membrane of red blood cells, which may lead to their rupture. However, no such changes were observed 24 h and 48 h after administration, indicating a possible transient effect [155]. Clinical trials conducted for intravenous infusion also showed a toxic effect in the blood. Patients experienced hemolysis and a decrease in hemoglobin concentration. A clinical study also noted a relationship between the occurrence of blood abnormalities and the dose of CUR [58]. Therefore, CUR liposomes should be considered for other routes of administration. Testing for solid lipid nanoparticles also did not show satisfactory results. The lack of tissue specificity resulted in the spread of nanoparticles to various organs, including the kidneys, liver, and pancreas. However, they showed low toxicity to human umbilical vein endothelial cells (HUVEC). Cell survival was around 80% [157].

In the case of polymer nanoformulations for CUR, those based on polylactic-co-glycolic acid (PLGA) did not show hemolysis [20,171]. For microscale discoidal polymeric particles at a dose of 25 mg/kg, in addition to accumulation in the lungs, which was the targeted organ, significant accumulations in the liver were observed. However, the formulation did not significantly affect AST, ALP, ALAT, creatinine, and BUN levels compared to the control group. This suggests that, despite accumulation in the liver, polymer nanoparticles caused no observable toxicity [171]. CUR-PLGA-PEG nanoparticles influenced reproductive performance in males. Short-term intravenous administration inhibited testicular cell line proliferation in mice and spermatogenesis. A harmful effect on the spermatogonia cell cycle and sperm motility was observed. Elongating spermatids caused a failure in spermacid maturation [172]. On the other hand, CUR-encapsulated alginate/Fe_3_O_4_ nanoparticles, when checking the effect of various doses, showed no acute toxicity. There were no performance behavioral changes in the mice, and none of them died after 72 h. However, the formulation influenced chronic toxicity. A slight weight loss and the death of two mice were observed over a longer period. Nanoparticles were present in small amounts in the liver and caused the vacuolization of hepatocytes and an increase in ALAT and AST biomarkers, indicating the possibility of liver toxicity [167]. The liver accumulation was also observed for the formulations of complex gold nanorods with CUR and mesoporous MnO_2_ nanoparticles with CUR [173,174]. Gold nanorods with CUR showed a negligible therapeutic effect resulting from a minor accumulation in the cancer tumor and a significant accumulation in the spleen. This triggered an inflammatory response that was minimized by the action of CUR. The formulation also harmed maintaining the proper functioning of erythrocytes [173].

## 9. Conclusions

Hepatic inflammation is a critical driver of chronic liver diseases, including MASLD and IFALD, which can progress to fibrosis, cirrhosis, and hepatocellular carcinoma. CUR exhibits potent anti-inflammatory, antioxidant, and hepatoprotective effects through various biochemical pathways. It reduces key markers of liver damage (ALAT, AST, and GGT), inflammation (TNF-α and IL-6), and lipid metabolism dysfunction, making it a promising therapeutic candidate for managing MASLD and potentially IFALD, which, despite differing etiology, share some similarities in the pathomechanism. However, CUR’s poor pharmacokinetics significantly limit its use as a therapeutic agent. To fully leverage its potential, its biodistribution must be enhanced through modifications such as nanoformulations, including nanoemulsions and cell membrane-derived systems, which have demonstrated excellent biocompatibility. While further research is essential to optimize CUR’s bioavailability and confirm its efficacy in liver disease treatment, the currently published studies provide promising evidence of its therapeutic potential.

## Figures and Tables

**Table 1:** The molecular targets for CUR.

Category	Targets	Reference
Transcription factors	NF-κB, Nrf2 *, AP-1, β-catenin, STAT3, HIF, Smad7, and Smad3	[22,23,24,25,28]
Growth factors	CCN2, EGF, FGF, HGF, NGF, PDGF, VEGF, and TGF-β	[23,24,25,28]
Kinases	PhK, PKCε, PAK, Pp60c-tk, EGFR kinase, CAMK, GSK-3β, AMPK *, and JNK	[23,24,25,28]
Inflammatory cytokines	IL-1, IL-2, IL-5, IL-6, IL-8, IL-10, IL-12, IL-18, MCP, MaIP 1α, TNF-α, and MMIF	[22,23,24,25,28]
Receptors	LXR, FXR *, keratinocyte transferring receptor, AR, AHR, CXCR4, EGFR, H2R, HER-2, IR, DR5 *, EPCR *, FR *, and PPAR-γ	[23,24,25]
Enzymes	ATPase, COX-2, LOX, SOD *, CAT *, GPx *, ODC, HAT, DNA polymerase, FPT, AATF-1, iNOS, NQO-1, PhpD, SRC-2 *, GST *, and HO-1 *	[22,23,24,25,29]

Most of the targets are inhibited or downregulated by CUR. *—targets stimulated by CUR. NF-κB: nuclear factor kappa B, Nrf2: nuclear factor erythroid 2-related factor 2, AP-1: activator protein-1, STAT3: signal transducer and activator of transcription 3, HIF: hypoxia inducible factor, Smad7: mother against decapentaplegic 7, Smad3: mother against decapentaplegic 3, CCN2: connective tissue growth factor, EGF: epidermal growth factor, FGF: fibroblast growth factor, HGF: hepatocyte growth factor, NGF: nerve growth factor, PDGF: platelet-derived growth factor, VEGF: vascular endothelial growth factor, TGF-β: transforming growth beta factor, PhK: phosphorylase kinase, PKCε: protein kinase C epsilon, PAK: protamine kinase, Pp60c-tk: pp60c-src tyrosine kinase, CAMK: Ca^2+^/calmodulin-dependent protein kinase, GSK-3β: glycogen synthase kinase-3 β, AMPK: activated protein kinase, JNK: c-Jun N-terminal kinase, IL-1: interleukin-1, IL-2: interleukin-2, IL-5: interleukin-5, IL-6: interleukin-6, IL-8: interleukin-8, IL-10: interleukin-10, IL-12: interleukin-12, IL-18: interleukin-18, MCP: monocyte chemoattractant protein, MaIP 1α: macrophage inflammatory protein 1α, TNF-α: tumor necrosis factor α, MMIF: macrophage migration inhibitory factor, LXR: liver X receptor, FXR: farnesoid X receptor, AR: androgen receptor, AHR: aryl hydrocarbon receptor, CXCR4: chemokine receptor 4, EGFR: EGF receptor, H2R: histamine H2 receptor, HER-2: human epidermal growth factor receptor-2, IR: integrin receptor, DR5: death receptor-5, EPCR: endothelial protein C receptor, FR: Fas receptor, PPAR-γ: peroxisome proliferator-activated receptor γ, COX-2: cyclooxygenase-2, LOX: lipoxygenase, SOD: superoxide dismutase, CAT: catalase, GPx: glutathione peroxidase, ODC: ornithine decarboxylase, HAT: histone acetyltransferase, FPT: farnesyl protein transferase, AATF-1: arylamine N-acetyltransferase-1, iNOS: inducible nitric oxide synthase, NQO-1: NAD(P)H:quinone oxidoreductase, PhpD: phospholipase D, SRC-2: Src homology 2 domain-containing tyrosine phosphatase 2, GST: glutathione-S-transferase, HO-1: heme oxygenase-1.

**Table 2:** Types of intestinal failure [72,73].

Type	I—Acute Condition	II- Prolonged Acute Condition	III—Chronic Condition
Reversibility of the primary disease	Reversible. Postoperative ileus and sudden intestinal obstruction.	Reversible. It usually occurs with unstable patients who may have suffered complications as a result of major bowel or any other surgery.	May be irreversible. Stable patients, who suffer from short bowel syndrome, surgical complications, or inflammatory bowel disease.
Duration of the PN therapy	Short-term PN (Days)	Short-term PN (Weeks or months)	Long-term PN (Months or years, in some cases lifelong)

PN: parenteral nutrition.

**Table 3:** Noninvasive diagnostics: physical features, biomarkers, and imaging techniques for active IFALD.

Type of Diagnostic Tool	Diagnostic Tool	Diagnostic Features	References
Physical feature	Physical examination of the patient data	Jaundice, hepatomegaly, and splenomegaly	[75]
Biomarkers	ALAT	Increased (>2–3 times the upper limit)	[95,101,102,103]
	AST	Increased (>2–3 times the upper limit) 44–302 U/L	[95,101,102,103]
	Bilirubin	Increased (2–3 times the pre-PN levels) 5.0–45 µmol/L	[95,103]
	Conjugated bilirubin	Increased (2–3 times the pre-PN levels) 2.3–28 µmol/L	[103]
	GGT	Increased 39–179 U/L	[103,104]
	Citrulline	Decreased 5.0–16 µmol/L	[103]
Imaging techniques	Transient elastography	Evaluation of liver stiffness	[103,105]
	Magnetic resonance spectroscopy	Evaluation of the degree of steatosis	[106]
	Proton MRS	Evaluation of the degree of steatosis (quantitative liver fat content)	[107]

ALAT: alanine aminotransferase, AST: aspartate aminotransferase, GGT: gamma glutamyltransferase, pre-PN: prior to parental nutrition, and MRS: magnetic resonance spectroscopy.

**Table 4:** Effect of different doses and formulations of CUR on the biochemical parameters and BMI occurring in MASLD, liver cirrhosis, and hepatic steatosis.

Formulation	Dose of CUR [mg/ Day]	Time [Weeks]	ALAT	AST	ALP	TNF-α	CRP	TGs	LDL	HDL	TC	FBG	IL-6	BMI	Ref.
CUR capsules	1000	12	N.S	N.S	N.S	x	x	x	x	x	x	x	x	x	[46]
CUR + piperine capsules	500	12	↓	↓	x	x	↓*	↓	↓	N.S	↓	↓	x	↓	[63]
Phytosomal/ Meriva^®^	50	8	N.S	N.S	x	x	x	N.S	N.S	N.S	N.S	N.S	x	↓*	[65]
Livogen Plus^®^	x	12	N.S	N.S	x	N.S	N.S	N.S	x	↑*	N.S	N.S	↓*	↓*	[66]
Amorphous dispersion of 70 mg of curcuminoids	x	8	↓	↓	x	x	x	↓	↓	↓	↓	N.S	x	↓	[142]
BIOCUR^®^	1500	12	↓*	↓*	x	↓*	N.S	x	x	x	x	x	x	↓*	[122]
Phytosomal/ Meriva^®^	200	8	↓	↓	N.S	x	x	x	x	x	x	x	x	↓	[150]
Nanomicelle	80	12	N.S	N.S	N.S	x	x	x	x	x	x	x	x	N.S	[151]
Phytosomal/ Meriva^®^	50	8	N.S	N.S	x	x	x	x	x	x	x	x	x	↓	[118]
Phytosomal/ Curserin^®^	200	8	x	x	x	x	x	↓	N.S	↓*	N.S	↓*	x	↓*	[133]
Phytosomal/ Meriva^®^	200	8	x	x	x	x	x	↓	↓	↓*	↓	↓*	x	x	[143]
BIOCUR^®^	1500	12	N.S	N.S	x	x	x	N.S	N.S	N.S	↓*	↓*	x	↓*	[134]
Phytosomal/ Meriva^®^	50	8	↓	↓	x	x	x	x	x	x	x	x	x	↓	[141]
Phytosomal/ Meriva^®^	50	8	N.S	↓	N.S	x	x	N.S	N.S	↓*	N.S	N.S	x	↓*	[144]
NanoCUR/ sinaCUR^®^	x	12	↓	↓	x	↓	↓	↓	↓	↑	↓	↓	↓	↓*	[137]
C3 Complex^®^ + Bioperine^®^	x	8	N.S	N.S	x	↓	x	N.S	N.S	N.S	N.S	N.S	N.S	↓	[139]

↓: significant decrease between treatment and placebo group, ↑: significant increase between treatment and placebo group, ↓*: significant decrease within treatment group, ↑*: significant increase within treatment group, N.S: non-significant differences, x: not measured, CUR: curcumin, ALAT: alanine aminotransferase, AST: aspartate aminotransferase, ALP: alkaline phosphatase, TNF-α: tumor necrosis factor, CRP: C-reactive protein, TGs: triglycerides, LDL: low-density lipoprotein cholesterol, HDL: high-density lipoprotein cholesterol: TC: total cholesterol, FBG: fibrinogen, IL-6: interleukin 6, BMI: body mass index, and Ref.: references.

## Abbreviations

The following abbreviations are used in this manuscript:

CUR	Curcumin
IFALD	Intestinal failure-associated liver disease
MASLD	Metabolic dysfunction-associated steatotic liver disease
NAFLD	Non-alcoholic fatty liver disease
MAFLD	Metabolic dysfunction-associated fatty liver disease
NASH	Non-alcoholic steatohepatitis
MASH	Metabolic steatohepatitis
NF-κB	Nuclear factor-kappa B
IL-1β	Interleukin-1 beta
DMC	Demethoxycurcumin
BMC	Bisdemethoxycurcumin
logP	Oil–water partition coefficient
t_1/2_	Half-life time
DMSO	Dimethyl sulfoxide
ROS	Reactive oxygen species
HO-1	Heme oxygenase 1
Nrf2	Nuclear factor erythroid 2-related factor 2
ALAT	Aminotransferase
AST	Aspartate aminotransferase
GGT	Gamma-glutamyl transferase
CRP	C-reactive protein
IL-6	Interleukin 6
ALP	Alkaline phosphatase
HRQoL	health-related quality of life
TC	Total cholesterol
HDL-C	High-density lipoprotein cholesterol
LDL-C	Low-density lipoprotein cholesterol
TG	Triglycerides
TNF-α	Tumor necrosis factor
TGF-β1	Growth factor-beta1
TβR II	TGF-β type II receptor
PN	Parenteral nutrition
IF	Intestinal failure
CYP7A1	Cholesterol-7a-hydroxylase
FGF19	Fibroblast growth factor 19
FXR	Farnesoid X receptor
LPS	Lipopolysaccharides
ILE	Intravenous lipid emulsion
LRH-1	Liver receptor homolog-1
ABCC2/MRP2	Multi-drug resistance protein 2
SHP	Small heterodimer partner
BSEP	Bile salt export pump
ABCG5/8	ATP-binding cassette transporters G5 and G8
LXR	Liver x receptor
PUFAs	polyunsaturated fatty acids
PPARα	Peroxisomal proliferator-activated receptor alpha
CPT1	Carnitine palmitoyltransferase 1
UDCA	Ursodeoxycholic acid
GLP-2	Glucagon-like peptide-2
NASH	Nonalcoholic steatohepatitis
SOD	Superoxide dismutase
AMPK	Activated protein kinase
JNK	c-Jun N-terminal kinase
MCP-1	Monocyte chemoattractant protein
EGF	Epidermal growth factor
IFN-γ	Interferon γ
VEGF	Vascular endothelial growth factor
8-OHdG	8-hydroxy-2′-deoxyguanosine
HbA1c	Glycated hemoglobin
GSK-3β	Apoptosis—glycogen synthase kinase-3 β
IAPP	Islet amyloid polypeptide
SIRT1	Deacetylase sirtuin-1
PGC-1α	Activated receptor-gamma coactivator-1 alpha
TLR4	Toll-like receptor 4
MyD88	Myeloid differentiation primary response 88
SLN	Solid lipid nanoparticles
HFn	Human heavy chain apoferritin
TPP	Triphenylphosphine
CXCR4	Chemokine receptor type 4
BUN	Blood urea nitrogen
PLGA	Polylactic-co-glycolic acid
SREBP-1c	Sterol regulatory element-binding protein 1c
BMI	Body mass index
ACC1	Acetylo-CoA carboxylase
STAT3	Signal transducer and activator of transcription 3
HIF	Hypoxia inducible factor
Smad7	Mother against decapentaplegic 7
Smad3	Mother against decapentaplegic 3
CCN2	Connective tissue growth factor
FGF	Fibroblast growth factor
HGF	Hepatocyte growth factor
NGF	Nerve growth factor
PDGF	Platelet-derived growth factor
PhK	Phosphorylase kinase
PKCε	Protein kinase C epsilon
Pp60c-tk	pp60c-src tyrosine kinase
PAK	Protamine kinase
CAMK	Ca^2+^/calmodulin-dependent protein kinase
MaIP 1α	Macrophage inflammatory protein 1α
MMIF	Macrophage migration inhibitory factor
MCP	Monocyte chemoattractant protein
AR	Androgen receptor
AHR	Aryl hydrocarbon receptor
CXCR4	Chemokine receptor 4
H2R	Histamine H_2_ receptor
HER-2	Human epidermal growth factor receptor-2
IR	Integrin receptor
DR5	Death receptor-5
EPCR	Endothelial protein C receptor
FR	Fas receptor
PPAR-γ	Peroxisome proliferator-activated receptor γ
COX-2	Cyclooxygenase-2
LOX	Lipoxygenase
CAT	Catalase
GPx	Glutathione peroxidase
ODC	Ornithine decarboxylase
HAT	Histone acetyltransferase
FPT	Farnesyl protein transferase
AATF-1	Arylamine N-acetyltransferase-1
iNOS	Inducible nitric oxide synthase
NQO-1	NAD(P)H:quinone oxidoreductase
PhpD	Phospholipase D
SRC-2	Src homology 2 domain-containing tyrosine phosphatase 2
GST	Glutathione-S-transferase
